# Defining and implementing social integration: a case study of school leaders’ and practitioners’ work with newly arrived im/migrant and refugee students

**DOI:** 10.1080/17482631.2020.1783859

**Published:** 2020-12-09

**Authors:** Osa Lundberg

**Affiliations:** Child and Youth Studies, University West, Trollhättan, Sweden

**Keywords:** Education, social integration, neoliberalism, `diversity management´, newly arrived, im/migrant, health, well-being

## Abstract

**Purpose:**

This study probes educational leaders and practitioner’s views about social integration with newly arrived im/migrant and refugee students. A sociological perspective of education is used in conjunction with a thematic analysis of neoliberal approaches to diversity management and its social implications for the health and well-being for im/migrant students.

**Methods:**

An interview study with 15 educational leaders and practitioners in schools and recreational centres was carried out. Thereof, seven department heads, three principals, and five educators. Data-production consisted of a semi-structured interview guide about practitioners’ views on social integration.

**Results:**

The results of the study indicate that there is a tendency to emphasize academic achievement and individual effort in compulsory education and in voluntary settings. The im/migrant students’ needs for help, assistance with social and psychological support are viewed as obstacles to social integration.

**Conclusions:**

Findings suggest universal approaches to diversity management in education tend to stress individual agency but fail to acknowledge individuals’ lack of control over structural factors. The organizational structure of schooling creates both affordances and obstacles for social integration beyond the control of the individual which add to the burden of social integration on the individual im/migrant students.

## Introduction

Newly arrived im/migrant and refugee students who are registered within a municipality in Sweden have the right to attend school; however, only students with citizenship or permanent resident status are obligated to attend compulsory schooling. Im/migrants and refugees with temporary resident status can attend compulsory school but are not obligated to do so by law. The Education Act (SFS 2010:800) asserts that purpose of education is to provide all children and youth with equal opportunity regardless of where they live to obtain knowledge, skills, values, personal development, and lifelong desire to learn. Despite these political and educational ambitions, sociological research of educational institutions claims that schools are sites of cultural reproduction of difference and disadvantage (Bourdieu & Passeron, 1990; Lundberg, 2015).

Although the Education Act (2010:800) and national curriculum state that the purpose of education is in part to promote social integration in schooling, there is no specific legislation on what social integration entails. This article takes issue with social integration discourses that emphasize economic inclusion and neoliberal ideology of individual choice and freedom (Levitas, 1997). The analysis attempts to unpack neoliberal values that impede rather than enhance opportunities for newly arrived im/migrant students’ ability to control the process of social integration and opportunities to further their own health and well-being.

This study examines school leaders’ and practitioners’ perspectives on social integration with newly arrived migrant students in a smaller municipality on the west coast of Sweden. More specifically, the data production concerns how school leaders and practitioners define and operationalize social integration within schools, leisure time centres and other municipally run cultural activities. The analysis discusses the way practitioners view the task of education in relation to the social implications of social integration on the health and well-being of im/migrant students.

The research questions are:

- How is social integration defined by educational practitioners? And how is this definition realized in the context of schooling and leisure time programmes?
- What are the social implications of practitioners’ views regarding social integration for im/migrant and refugees’ student health and well-being?

## Literature review

### Neoliberalism and education

In this section, I will present and discuss studies on neoliberalism and its impact on im/migrant and refugee students with regards to transitioning practices into education in host countries. Some contemporary issues and themes include educational leadership, intercultural pedagogy, and racialization of im/migrant students of colour. Of interest here are previous studies concerning stakeholders such as school leaders’ and educators’ involvement in recontextualizing policy and how these practices limit the degree of control migrant and refugee students have within these institutions to practice social integration.

Previous studies illustrate how neoliberalism is imbricated in policy management and implementation regarding the education with im/migrants. In the Swedish context, Norberg’s (2017) study on educational leadership and im/migration examines the lack of training and preparation school leaders receive with regards to managing diversity. Norberg asserts that there is a gap between educational initiatives to create equity and equality and the way schooling is implemented with im/migrant students. Respondents in Nordberg’s study emphasized the overall task of schooling as promoting students learning and achieving social and academic goals. However, school leaders also discussed the need for increased intercultural competence amongst teaching staff and the need to combat norms of Swedishness and deficit-perspectives of non-native im/migrant students (Norberg, 2017). Norberg’s study indicates a conflict between the neoliberal values for the right to education for all students and the difficulty for school leaders and educators to see how academic goals and standards of achievement ignore the diversity and disadvantaged position im/migrant students.

Equity and equality in the neoliberal framework are often expressed in terms of individual freedoms, choices, and decisions. Hertzberg (2014) found that career guidance counsellors emphasized im/migrant students’ autonomy and ability to make informed decisions as a means to societal inclusion. Im/migrant and refugee students who could not make informed, independent autonomous, decisions were deemed to be self-selectively excluded because of their inability to conform to standards of autonomy and self-assertion. Social inclusion in the neoliberal framework is often reduced to traits or characteristics within individuals who are expected to access knowledge and take initiatives on their own accord. These traits, independence, and autonomy are not merely assigned to individuals but are also reflective of a collective consciousness and ideals regarding citizenship, ethnicity, and identity of the dominant group. The respondents in Hertzberg’s study did not assign autonomy, reason, and rationality to conservative ideals of culture or ethnicity; liberal ideals of equality and freedom were seen as universal rather than culturally specific (Hertzberg, 2014). Furthermore, Hertzberg (2017) observes that collectivistic values, as opposed to ideals of individualism, are regarded as erroneous and incompatible with educational norms. The conclusion that collectivistic values are incongruent with neoliberal norms of individualism and independence is similar to findings in other studies (Rah et al., 2009). Collectivistic values are viewed as erroneous and incongruent with neoliberalism which values autonomy and independence above family involvement, community control and collectivism (Hertzberg, 2017; Jaffe-Walter & Lee, 2018; Mac an Ghaill & Haywood, 2014; Rah et al., 2009).

Previous research findings also assert that assimilationist and acculturation perspectives continue to be persistent and pervasive; more inclusive policy and practices call for the continued development of mother tongue languages for transnational students (Evans & Liu, 2018; Jaffe-Walter & Lee, 2018). Inclusive pedagogical approaches to social integration assert the identity development and prior knowledge of students’ cultural and linguistic capital as a means of adaptation and inclusion to the host country and increase opportunities for students to develop transnational ties to their home countries in the future (Jaffe-Walter & Lee, 2018). Research shows that increased collaboration with parents and immigrant communities can facilitate students’ academic success (Rah et al., 2009). Rah et al., 2009 and Reath Warren (2017) found that Vietnamese refugees benefitted academically from educators’ involvement and collaboration with parents and the refugee community. This type of facilitation of learning that builds on students’ prior knowledge and promotes collaboration with im/migrants’ families and communities is costly and not readily reduced to measurements of academic achievement.

International research on social integration indicates the importance of schooling and the necessity to assist newly arrived students in building positive social relationships with teachers and peers. Ron-Balsera (2015) study of Ecuadorian migrant youth in Spain discusses how the role of peer friendships and supportive relationships with teachers enables migrant students to stay in school and withstand exclusion, aggression, and marginalization. Intra-ethnic friendships with other non-white and Ecuadorian students were more commonplace than with native-born Spaniards. Teachers did not seem to concern themselves with bridging inter-ethnic ties or to address micro- and macro-aggressions such as racism, sexism, and classism (Ron-Balsera, 2015, p. 169). Similarly, in a study of Pakistani and Bangladeshi young men in England, Mac an Ghaill & Haywood (2014) found that the neoliberal logic resisted community and family interdependence. Educators’ promoted individual responsibility, entrepreneurialism, and competitiveness. These values were endorsed by white middle-class teachers. The young Pakistani and Bangladeshi men associated the entrepreneurial self and individualized choice with normative whiteness and middle-classness (Mac an Ghaill & Haywood, 2014, p. 766). Pedagogical practices that endorse neoliberalism function to create citizens with knowledge that can be commodified for a global market and “such ideologies of citizenship tended to hide the racialization of class differentiation” (Mac an Ghaill & Haywood, 2014, p. 767).

In sum, previous research indicates that the logic of neoliberalism operates through promoting the values of independence and autonomy as universal values and placing a heavy focus on academic achievement as the means to social integration, while downplaying the role of community and family ties. Im/migrant groups that value collectivism and interdependence are not only at a disadvantage in liberal democratic schooling structured on individualism but are also seen as resisting social integration and upward social mobility (Hertzberg, 2017; Mac an Ghaill & Haywood, 2014; Ron-Balsera, 2015). In this article, I argue that there is a discrepancy between the aims and ideals of neoliberal values embedded in the way the purpose of education is expressed and the needs of the im/migrant students that extend beyond economic inclusion (Levitas, 1997; Lister, 1998).

### Illiberal liberalism, “diversity management”, and the myth of equality

Sweden has long held a position of exceptionalism with regards to egalitarianism, openness and tolerance towards im/migrants and refugees (Schierup & Ålund, 2011a). In the 1960s and 1970s liberal integration policy and legislation guaranteed migrants and other immigrant groups the rights to work, welfare and citizenship. However, Sweden in more recent years has lost some of its exceptionalism due to neoliberalism and the call for tighter controls of migration (Schierup & Ålund, 2011a). Neoliberalism which emphasizes individual’s rights and freedom of choice has transpired into what Schierup and Ålund (2011b) refer to as *illiberal liberalism* in which the responsibility of integration is put upon the im/migrant to culturally assimilate and to make one’s self available to the labour market by way of education and acculturation. The inclusion of im/migrants is made possible in the liberalist framework by the social provisions and availability of education for all. Education for all in Sweden is also a dimension of the Swedish labour market policy to mitigate unemployment. In this sense, it can be argued that social integration is often reduced to economic inclusion (Levitas, 1997; Lister, 1998)

The concept of `*diversity management´* displaces the previous focus on human rights, equality, and identity with a focus on effectivity in policy implementation in institutional and organizational practices. This neoliberal effectiveness of social and cultural diversity downplays difference and emphasizes universal rights and responsibilities as conditions for inclusion in liberal democratic societies (Mouffe, 2000). Diversity management that creates the provisions for inclusion also has well-defined objectives such as being available to the labour force and/or enrolled in education. Neoliberal management of cultural diversity is, in this sense, one of efficiency and skirts the issues and messiness of multiculturalism (Noble, 2009). Neoliberal strategies tend to avoid antagonism, downplay resistance strategies of marginalized groups, and promote education as a universal equalizer of social and economic disparities. Universalism, in the neoliberal framework, becomes an efficient way of managing and dealing with diversity and opposition under the guise of equity.

The myth of equality (Ladson-Billings, 2006; Wellman, 1993) in education is reflected in the belief that all can make it if they try hard enough. The myth of equality is a belief in meritocracy in which a person’s social standing is due to his/her goals and accomplishments. This belief ignores structural forms of discrimination that occurs in the labour and housing markets and other institutional factors that obstruct conditions for inclusion (Andersson et al., 2009; De Los Reyes, 1998, 2000). The myth of equality is a pervasive and persistence belief in the educational system which measures social inclusion by merits and academic outcomes. The myth of equality works to perpetuate inequalities in the educational system based on the underlying belief of school knowledge as universal and objective (Lundberg, 2015; Mills, 1997; Willhelm, 1998). Myths have a tendency to become reality when for all intended purposes people believe and adhere to them (Bloodworth, 2016; Lareau, 2011; Meyer, 2007).

Neoliberalism promotes efficiency, effectivity, and individual choice. Neoliberalism has usurped integration principles of equality, freedom of choice and partnership that were previously inscribed in a framework of multiculturalism (De Los Reyes, 2000). The multiculturalist framework is now displaced by management driven policy of diversity. Diversity management in the neoliberal framework has redirected the focus of social integration from discourses of inequality and poverty into `*equality of opportunity*´ with a strong emphasis on economic inclusion and sameness approach to diversity and cultural difference (Hertzberg, 2014; Lister, 1998; Schierup & Ålund, 2011b). All children and youth can attend compulsory schooling within their local municipalities in Sweden. Economic inclusion is afforded by state-financed compulsory schooling for im/migrant school-age children, even those who are not registered and do not have official legal status as residents (Government [Regeringen], 2019).

In this study, the key analytical concepts presented here are used to critique the impact of neoliberal values on newly arrived migrant and refugee students’ health and well-being.

## Methodology

### Site selection

The case in point is a smaller municipality on the west coast of Sweden with a population of 29 000 inhabitants and foreign-born population of 15% in 2016. This site was selected because of a new law on resettlement that required all municipalities in Sweden to make provisions for newly arrived migrant children and families with residence permits since the introduction of the Settlement Act (Act, 2016:38). The purpose of the settlement act is to provide newly arrived migrants and refugees the opportunity to become part of society and the workforce. According to the County Administrative Board recommendations, this county received 98 migrants/refugees in 2016 after the arrival of large numbers of refugees to Sweden in fall of 2015, many of which were unaccompanied minors.

### Research participants

The empirical data production includes 15 interviews with 7 department heads (including project leaders), 3 principals and 5 educators. The department heads worked in language development, recreational and cultural programmes, plus a leader in a municipally sponsored project for social integration. Two principals worked in compulsory education and one in upper secondary. The educators interviewed worked in compulsory, upper secondary and recreational programmes with all children and youth, not just migrants and refugees. This study takes a leadership and practitioner perspective on the settlement and social integration within schools and recreation centres geared towards child and youth leisure time activities. The interviews include questions on their views of social integration within the context of educational, cultural and recreation programmes for children and youth.

### Semi-structured interviews and data analysis

The interviews were carried out in person and took one hour each. Thereafter, the interviews were transcribed, coded, and analysed thematically with the aid of NVivo software for qualitative data analysis (Clarke & Braun, 2017). The analysis involved an iterative process from transcription of initial interviews, coding, aggregation of codes into themes and summarization of themes. The coding and construction of themes were informed by the research participants’ responses, previous research and conceptual framework in a constant comparative approach and thematic analysis (Boeije, 2002; Clarke & Braun, 2017; Glaser, 1965). The primary theme discusses how school leaders and practitioners conceptualize and operationalize social integration with regards to their professional roles to the overall task and purpose of education and how these views and processes can be understood in relation to a neoliberal framework.

## Results

### Academic achievement

The data analysis show that the research participants view social integration as becoming part of society. In their role as professionals, the research participants discussed this view of social integration in relation to the primary task and purpose of education. The purpose of schooling according to the respondents is to educate students.

What is your job assignment?

(Osa)

In relation to newly arrived students? It is about providing them with an education so that they can continue [building on] elementary competency. [So] that they can continue to high school and out into the workforce. And the change we have seen over the last few years I believe we will broaden. Previously it was only Swedish as a second language, but now includes all other subjects.

(Principal 3)

In the excerpt above Principal 3 refers to the importance of competencies and skills necessary for employability. Newly arrived students are given the opportunity to acquire official knowledge and subject matter. The principal here describes the task of education in terms of academic achievement and workforce preparation. This view is reflective of the Swedish labour policy for education to supply future workers for the workforce. Embedded in the educational task is a supply of independent, autonomous, and competitive individuals for the labour market (Levitas, 1997; Lister, 1998). However, workforce preparation and employability is only one solution amongst many other hurdles and more immediate obstacles to social integration facing im/migrant students. As we shall see, this emphasis on education as the means to social integration places undue emphasis on economic inclusion (Levitas, 1997; Lister, 1998) at the expense of im/migrant and refugee students’ social and psychological needs.

The compensatory pedagogical task of education can be divided into academic knowledge and social needs. The social needs are regarded separately and are kept apart from the primary task of education, which according to the research participants is to first and foremost provide all students with an education.

As I said, there are housing problems, unemployment problems. That is not the school’s problem. When we have succeeded in integrating newly arrived students into the Swedish school we have succeeded with integration into society.

(Department Head 1)

In the preceding quote, the Department Head 1 equates social integration with education and learning. Aligning education with social integration is reflective of neoliberalism’s universal approach to managing diversity by focusing on individual academic achievement. Teachers are aware of newly arrived students’ needs and concerns with housing and employment; however, subject matter and language acquisition are foregrounded as the core assignment and primary task of education. It is assumed that education is a precursory condition for future employment. The department head 1 implies that in “we have succeeded with integration into society” when educational requirements are met. The students’ immediate needs for work and housing support are expressed as, “not the school’s problem”.

You forget that school is not supposed to be a treatment center. It should be a school. Just so students can get a break from everything. They live in their lives the whole time and go through hardships. You do not need to reinforce this in school, instead you can reinforce your learning and progression. Your development. Each time you repeat something it sticks a bit better until next time. That is how it is. We have lost the focus that this is a regular school.

(Teacher 3)

Teachers and other practitioners are aware of the compounded and complex needs of newly arrived students. Teacher 3 recognizes the hardships of newly arrived im/migrant students and their need for social and psychological support. Yet, the compensatory measures taken to assess and accommodate these needs are foregrounded in terms of academic rather than social or psychological needs. In the neoliberal framework, academic success is defined primarily as an individual rather that collective process.

Neoliberal values are reflected in the universal access and availability of education for all. However, this universal approach to social integration does not adequately address the social and psychological needs of newly arrived im/migrant students (Hertzberg, 2017; Rah et al., 2009). A universal supply and access to education manages to paradoxically turn a blind eye to social differentiation and disadvantaged (Lareau, 2011; Mac an Ghaill & Haywood, 2014; Norberg, 2017). It can be argued that the educational task of supplying the labour market with workers pushes more pressing social issues of “treatment” for social and psychological concerns to the roadside. The more immediate concerns for housing and employment are viewed as secondary to the primary task of schooling.

### Criteria for inclusion

Research participants describe the process of social integration into schools as an administrative and organizational action in which newly arrived students make a successive transition from temporary reception classes and/or Swedish as second language programmes into other ordinary mainstream classes and programmes. In compulsory schooling, students have some influence over the pace of transition. School leaders take into consideration students’ prior knowledge and individual desires in the transition process. There is no one uniform model of reception for newly arrived students. It is up to the discretion of individual heads of schools to interpret and implement guidelines for reception (Skolinpektionen, 2017; Skolverket, 2016).

It depends a lot upon what kind of school background they [newly arrived students] have with them. We have students who have been in a reception class one semester and then go into an ordinary class. And we have students who have been in a preparatory group and needed to transition slower because they came here as illiterate and needed much more reading practice. They have very different preconditions. It is difficult to say how long students are in a preparatory group.

(Teacher 2)

It is always on an individual basis and we look to the screening of how much prior school experience a student has.

(Principal 1)

In the neoliberal framework, there is a strong emphasis on individualization, what the individual knows and his/her academic career. The mapping and assessment practices help to determine the placement of newly arrived students and the pace of integration from preparatory classes into mainstream classes. Principal 1 asserts, “It is always on an individual basis”. The emphasis on individualization can be seen as a reflection of neoliberal values of individual choice and freedom (Levitas, 1997). This individualized and universal approach to diversity management is an effective means of skirting the issues and messiness of multiculturalism (Noble, 2009). The compensatory task of education in the neoliberal framework is to compensate for deficits in official school subject matter.

It is decided that we should use the Swedish National Agency for Education mapping materials. These exist. We map the first parts, general school background, language, literacy, numeracy and then the agency has also added other subject that schools can continue with. There is English, mathematics, science, and some for Swedish. Schools are working hard with these issues.

(Teacher 2)

Here, the mapping of individuals knowledge and skills highlights the students’ strengths and weaknesses in literacy and numeracy. These are the two main fields of knowledge that are assessed and become the basis for placement and transitioning into the school system. Mapping, assessment, and placement of individuals according to their age and prior knowledge are reflective of individualization, but it can also be argued that these are structural and organizational aspects are beyond the control of the individual. Individualization may seem fair in a neoliberal framework, but it also implies that individual skills and knowledge provide the basis for inclusion and criteria of acceptance.

### Separate programmes

The upper secondary school is organized on separate but equal model of education. Students in elementary school have more opportunity to participate and influence the structure and organization than upper secondary students. A principal for upper secondary school explains the segregation and separation of im/migrant and refugee students.

The compulsory school works with preparatory groups that also study together with peers in mainstream classes. Newly arrived students in upper secondary school cannot not do so. Integrated classes can be arranged. We are feeling our way but have not come so far yet. We have music education, PE and health, outdoor activities and study visits, and other things with groups occasionally. We have discussed swimming. Our Swedish students can usually swim. A few students from the Language Program also attend sometimes. We are trying to go in that direction with the Individual Program, but there are students in that program [Individual Program] that also have great need for support in other ways. There are students with cognitive and psychological difficulties, or socially disadvantaged [in the Individual Program].

(Principal 3)

These students are kept separate and apart from mainstream classes and have little or no opportunity to socialize with other adolescents. All the national programmes in science, social studies, children and recreation, healthcare aesthetics and technique in the upper secondary school were discontinued because of economic reasons. This decision was made by the municipal government in 2013 and reflects patterns of separate but equal approaches to integration of im/migrants students in similar studies (Bunar, 2010; Stretmo, 2014). Providing education for newly arrived im/migrant students while keeping them in separate programmes may suffice the political education and labour market policy mode of integration but does little in terms of providing for their “psychological difficulties” or the “socially disadvantaged”.

Since the closure of the national programmes, only two programmes remain: The Language Programme and the Individual Alternative Programme. The Individual Alternative Programme is directed towards students with special needs and provides prerequisites for continued studies and the labour market. The Language Programme provides newly arrived students with knowledge of Swedish and compulsory education requirements. Both programmes span 1–3 years and are directed towards socially disadvantage groups in need of remedial education and/or special needs.

We are not prioritized as a school, neither of the programs are. We are not included in the discussion on schooling. It is clear that the student groups we have are not the priority groups.

(Teacher 3)

The Individual Alternative Programme and the Language Programme have a low status and priority within the municipality. Both the Language Programme and Individual Alternative Programme have students that can be categorized as disadvantaged and in need of compensatory measures to obtain learning outcomes for compulsory schooling and prerequisites for further education. Newly arrived students’ social status is embedded in the school organization and political structure. Teacher 3 indicates that the im/migrant students are a low priority group. The teachers are aware that being categorized as a low priority adds to the distress and hardship of im/migrant students.

The students are segregated and grouped by what they cannot do and what competencies they lack. Again, the conditions for inclusion are based on individual knowledge and skills. This is a structural and organizational decision made at the meso-level of school organization.

A few students have it really good. They have come here and established themselves and found contexts that they enjoy and work well in. But I have to say the majority experience a great exclusion and they do not enter society. They lack contact with Swedish youth. Many carry a great amount of anxiety and worry for their future because they do not know if they can stay or not. This influences their opportunity to make contact. You are not as outgoing when you have such an anxious situation as these youths have today.

(Principal 3)

In the quote above, newly arrived students both promote and obstruct their own integration. Principal 3 claim they obstruct integration because of worry and uncertainty about their future. In this logic the individual is an obstruction to his-/herself. This demonstrates the illiberal side of neoliberalism when students are to blame for their own inability to acculturate into the dominant culture. The deficit perspective on individuals’ mental health and knowledge obscures the institutional and organizational structures that create the criteria for inclusion. The im/migrant students’ health and well-being is a prerequisite for social inclusion according to Principal 3. Foregrounding the individual perspective tends to obfuscate the role of institutional organization by imposing the responsibility of social integration on the individual obscures.

Concomitant with the focus on knowledge and learning is a discourse of care. Care for newly arrived students’ psychological and mental health is expressed through the discourses of anxiety and worry. These parallel discourses reveal tensions in the way the official assignment of schools is interpreted. An emphasis on knowledge and learning is seen as a reprieve from mental stress. Yet, isolation and exclusion from society are additional stress factors that compound newly arrived students’ anxiety. On the one hand, the informants construct im/migrant students as being in psychological distress; on the other hand, “treatment” (Teacher 3) is not the primary task of education. The im/migrant students’ current social and psychological needs are secondary to the future aims of providing educated workers. At the same time, the uncertainty of im/migrant students’ future is acknowledged as a primary stress factor.

### Educators facilitate social integration

Social integration is discussed in terms of the school’s assessment of prior learning, as mentioned above, and in terms of direct integration to mainstream classes. Direct integration into classes occurs in some schools in which there are no preparatory classes for newly arrived students. Direct integration is the preferred choice for social integration in schools in which there are a relatively few and very heterogenous group of newly arrived students admitted intermittently throughout the school year. The newly arrived students vary with regards to ages, language, ethnicity, nationality, prior school experiences and begin classes spanning from reception/kindergarten to grade nine. Newly arrived students are therefore permitted to attend schools and become directly submerged in learning with their peers in mainstream classrooms. Other schools in the municipality have opted for other forms of organization in which newly arrived students attend a preparatory class first and are admitted to a mainstream class twice during the school year (fall and spring semesters). In contrast, direct integration affords opportunities for the mainstream class students to increase their awareness and practice of social inclusion on a continual and intermittent basis throughout the school year.

How does social interaction work between established Swedish students and newly arrived?

(Osa)

When we place a student, we often discuss this with the students in the class, both Swedish students and students from other cultures. We talk about how important it is to take care of new classmates. That there is a new classmate who can communicate in English. Or can communicate a little in Swedish and their mother tongue. That it is important to take care of, invite in, and show consideration. A new student doesn’t go up to others and say,” Can I be with?”. Instead you have to be the one to invite them. That is what we talk about before a new student starts here. Of course, you learn things along the way. Newly arrived students can be quite direct, they are not shy. Sometimes they are quiet and shy. Then you need to invite them in, ask questions and check with them, “How are you?”

(Principal 2)

In the quote above, Principal 2 discusses the role of the school leadership and teachers to ease transitioning into the school. This principal discusses care and concern for the individual from the class perspective with the emphasis that there is a shared responsibility and mutual concern for the welfare of the newly arrived students and inclusion by their peers into a new class. The point here is that leadership and teachers help construct a collective awareness amongst mainstream students to acknowledge and adapt to students in a marginal position.

The newly arrived students from Afghanistan who arrived as unaccompanied minors and with their guardians experience different degrees of inclusion and exclusion.

The boys from Afghanistan have expressed a desire to meet established Swedish people but have difficulty doing so. I was wondering how it is here, as you describe it, it does not seem that way.

(Osa)

No, we have three newly arrived students from Afghanistan. They do not socialize with each other. They have their own friends. We have talked about that. About these three boys. They are very eager to have their own friends. Two live with a foster family but do not socialize together here. They have their own friends, and all have new contacts. It is my experience that they are very accepted. All the students are.

(Principal 2)

With regards to direct integration, the boys from Afghanistan assimilated directly into the ordinary classes without difficulty according to Principal 2. However, im/migrant students’ health and well-being socially is not simply a matter of individual effort. The im/migrant students discussed here by Principal 2 are dependent on the school leaders and educators to facilitate social integration in schooling. Direct integration affords opportunity for social integration with newly arrived students and their peers. However, these affordances stand in direct relation to the degree of involvement of mother tongue and study guidance tutors.

I am the link between the child and the nurse, curator, special education teacher, mentor, and principal. This means a lot more work that just knowledge. I am the link between all these people.

(Teacher/pedagogue 1)

As indicated in the quote above, mother tongue teachers and study guidance tutors act as bridges between other students, teachers, parents, and principals. The study guidance tutors communicate information between different partners and provide ongoing assistance and guidance for newly arrived im/migrant students and their families. The newly arrived students and families are dependent upon and employ mother tongue teachers to facilitate social integration. By extension, these educators also facilitate the health and well-being by extending help beyond their role and task as language and/or study tutors. This indicates that the organization of compulsory and voluntary education is beyond the control of the individual. I argue that im/migrant students’ health and well-being is not just a consequence of individual agency but also dependency on educators’ assistance with social integration. Newly arrived im/migrant students are dependent upon the study support provided by school leaders and practitioners to facilitate communication by voicing the needs of im/migrant students.

### Im/migrant students’ lack of control

In compulsory schooling, all activities are to a greater or lesser extent required. Elementary school is mandatory and there is a lack of voluntariness and free will. According to the research participants, voluntariness and the absence of coercion are precursors for real and genuine meetings and relationships.

As soon as you go somewhere, you do so to get a grade or credit, then it is no longer voluntary. Your focus is somewhere else. The whole concept is difficult. But obviously you can create social things too.

(Teacher 2)

In the excerpt above Teacher 2 indicates academic achievement as the primary motive of education, not social interactions, or relationships per se. As mentioned before, this underlines the main task of education as preparation for the workforce. Teacher 2 adds, “But obviously you can create social things too.” Voluntariness reduces social integration to a byproduct of academic achievement, not a primary target or goal of its own.

A place of social integration outside of school is “The Mill”. All the research participants mention The Mill as a voluntary meeting place for all newly arrived im/migrant and refugee students and families in the municipality. The Mill provides opportunities for newly arrived youth and families to meet other residents, speak Swedish and make friendships. The purpose of The Mill is to build relationships and bridges between the newly arrived and other long-standing residents.

All newcomers get a contact with a Swedish resident and vice versa. We support and encourage this. That there is a Swede for each newcomer.

(Project Leader)

The Mill offers a language café and tutoring for students who want to improve their language skills and knowledge in Swedish and other school subjects. The Mill is seen as free from obligations, learning outcomes, subject matter teaching, assessment and other activities that characterize formal learning in the educational system. Although The Mill is cited as a voluntary meeting place, free from obligations, the activities are geared towards language acquisition in Swedish and learning of school subjects.

What we do for youth is to be a meeting place for young people. It is not a cultural activity center. There is a language café every Tuesday from 17:30–19:30. We even offer help with homework. Those who want homework assistance to get help through cooperation with the housing staff. They [newly arrived students] always have a contact person available who see that you need advanced homework assistance in Swedish and they have a plan of action as well.

(Project Leader)

Even the voluntary arenas work to support the learning outcomes of compulsory education. Regardless of whether newly arrived students are in school or outside of school, social integration tends to focus on school knowledge and promoting academic achievement. The project leader states, “We even offer help with homework.” This indicates that the voluntary social arenas assist newly arrived students with compensatory measures towards furthering academic rather than social needs. The well-being of the im/migrant student is secondary to the long-term goal of employment and labour market policy. Again, I argue that it is not the individual who is in control of social integration. Even in the so-called voluntary social arenas, it is the organization and structure of the social arena that controls the function and purpose of these spaces for im/migrants and refugees.

### The burden of social integration

In the municipalities’ cultural and recreational activities there are a score of activities and resources available for children and youth. Within the department of recreation, newly arrived youth can plan and organize activities with peers on their own initiative. The department of recreation provides places and leaders (youth coaches) who support young people’s plans to arrange social activities. The cultural and recreational department have no particular plan of action or measures directed towards newly arrived migrants or refugees; rather, these so-called meeting places are available to all youth regardless of social background.

We do not have any special projects for newcomers in that way. What we have seen is that they want to integrate and have very high ambitions. They desire a lot. In our activities all are welcome. Everyone works so that all will want to take part.

(Department Head 2)

In this quote, the Department Head 2 infers that “all will want to take part”. The cultural and recreational provisions construct an equality of opportunity in the form of accessibility and availability for all. However, as we shall see, the equality of opportunity does not mitigate inequalities in social status and relations of power beyond that lie beyond the control of im/migrants.

My role as youth coach in this municipality is to stimulate activities that build on and presuppose young people’s own active engagement and responsibility. That is the role.

(Department Head 2)

Help to self-help?

(Osa)

Precisely. That is our assignment from our politicians. We have the task from our sectional leader from the department of culture, recreation, and education; the overriding task is the desire to learn. There are several entries. If you understand the usefulness of knowledge, you have the desire to learn. For example, if you have understood how to book a bus and count the number of passengers. Small elementary things. All the while you know what you have accomplished and what you are good at. You get knowledge, which is good. We hope that we can generate the desire to learn even in school and that there is an increased understanding about it.

(Department Head 2)

The recreational department presupposes that children and youth can decode this kind of self-regulation, free choice, and the view of knowledge construction by way of self-initiative. The practitioners are conscious that these premises are unfavourable and unfair for socially and poor students. The thresholds to self-regulation, self-actualization, and realization of self-help initiatives to learning are many. Research indicates that working-class children and youth can find it difficult to decode and orchestrate their own learning (Bernstein, 2000). The voluntariness and self-initiative involve levels of self-mastery, independence, and social networking that im/migrant students may certainly behold but have not yet accrued in the local Swedish context. Furthermore, other studies indicate that values of interdependence and collectivism within im/migrant groups are seen as resisting integration (Hertzberg, 2017; Jaffe-Walter & Lee, 2018; Rah et al., 2009). While being a necessary resource, interdependence within the same ethnic group may be seen as counterproductive. However, the mainstream youth are neither inclined to seek contact with ethnic minority youth nor are they burdened with the responsibility to socially integrate.

The practitioners in the recreational programmes do not have a mandate to make targeted interventions; they do not seek out newly arrived students or any other socially disadvantaged group. There are no compensatory measures to counter inequality between youth with strong and weak social networks. Self-initiative is a prerequisite and principle regardless of social position. One research participant discussed the thresholds and contradictory conditions for newly arrived students’ opportunities to gain entrance and become a part of society:

There are obvious barriers, clear obstacles, and differences. It was revealed that when newly arrived boys arranged something. We had for example, an Afghani dinner in February. Two boys made kabuli and invited everyone. Both Swedish and Afghani youth were welcome, all young people. There were 27 Afghanis and 5 Swedes. That was during Easter break. Two other boys from Afghanistan arranged a Piñata party and the same thing happened again. Forty boys, all newly arrived and five Swedes. It was very difficult to get the Swedes to attend. That is where we have an issue in our team because it goes against our directives to influence. At the same time, you want to intervene and help.

(Teacher 4)

Insufficient interest amongst the youth in the municipality to participate in social activities initiated by the newly arrived youth obstructs social integration. The practitioners assert that the newly arrived youth make many attempts to invite and include mainstream youth. For example, the newly arrived students arranged a pool party and dinner party for girls. These activities were poorly attended by so-called Swedish youth.

In principle, these activities are open invitations with a theme, activity, or hobby that young people arrange on their own with some assistance from the recreational leaders. Yet the newly arrived students who make the effort to actualize the opportunities afforded to them in these meeting places cannot overcome the indifference of mainstream youth. Neither can they influence the practitioners’ directives not to intervene, take initiative on behalf of any youth group, or initiate any form of targeted outreach with privileged or disadvantaged groups.

Disinterest by majority youth is a factor beyond the control of newly arrived im/migrant youth who attempt to make the effort to socially integrate within the existing social structures. Socially disadvantaged students lack the cultural capital in a currency that is deemed valid by the dominant culture, to attract students with higher social status. Upward mobility is desirable, downward mobility is not. Constructing voluntary meeting places ignores dimensions of self-segregation based on status, cultural capital, racism, and a desire for upward, not downward mobility (Ron-Balsera, 2015). I argue that this is an illiberal consequence of liberalism in that social integration organized on a self-initiated and voluntary basis is obstructed by factors beyond the control of individuals.

## Conclusions

### Definitions and realizations of social integration

In the respondents' views, social integration is discussed in terms of academic achievement, Swedish language acquisition, and educational competence as the general solution to integration. In its efforts to provide equality of opportunity, the compulsory school strives for all students to obtain educational competencies and compensate for social and cognitive deficits. Nonetheless, the respondents’ views of social integration are ambiguous and require a much broader (re)definition with regards to newly arrived students’ needs for security, friendships and practical assistance that cannot be reduced to grades and subjects. I argue that neoliberalism feeds into cultural blindness that constructs social and academic goals as universal and inhibits the way these are culturally situated and embedded in local and particular modes of conduct and moral imaginations (see Bernstein, 2000).

The organization and structure of voluntary and compulsory programmes tend to be barriers to social integration beyond the control of the students. For example, it is my position that the organizational structure of upper secondary schooling highlights the illiberal aspects of neoliberalism in which students are organized into groups based on deficits of language and school knowledge. Worse yet, this organizational structure runs the risk of exacerbating the social exclusion and inadvertently adding to the mental stress and anxiety of im/migrant and refugee youth. The emphasis on academic and economic inclusion can obfuscate the practical, social, and psychological needs.

Furthermore, the educational system articulated by research participants is viewed as separate from sites of authentic and voluntary social integration. This logic of separateness and genuine social integration raises several questions. Can the educational system afford to prioritize long-term labour policies at the expense of im/migrants’ more immediate needs for social inclusion? And is there such a thing as voluntary social integration? School, work, and public spaces all require social integration to some degree. Even in voluntary social arenas students’ language skills and school knowledge are under scrutiny. These tendencies reinforce neoliberal ideals in which formal and informal learning contexts put a premium on individual agency and effort as the means to social integration and a universal approach to diversity management.

### Social implications of social integration discourses

Despite the research participants’ concern for the newly arrived students’ assailable situation and desire for contact with their peers in the community, the school articulated as having limited resources to promote social integration that falls outside the core task of education. The structure and organization of schooling is constructed on the discourse of learning, knowledge building and academic outcomes. The universal approach to education avoids the messiness of multiculturalism by creating a neat and tidy version of diversity management that is perceived as equitable, fair, and just.

Diversity management in the universal approach to education (accessibility and availability) disregards inequalities of power, such as control over the processes of social integration, im/migrant students’ ability to compensate for their low social status and to obtain cultural capital that is recognized and validated by the dominant group. There is an underlying assumption and expectation that newly arrived im/migrant and refugee students will take initiative and utilize formal and informal social arenas to promote social integration on their own. This puts an undue burden of social integration on the shoulders of im/migrant students who are already at risk for social and psychological stress factors concerning housing, employment, and dependency on others to facilitate the process of social integration.

Individual choice and freedom cannot mitigate or bypass structural obstacles such as segregation of newly arrived students, aversion to downward mobility, deficit perspectives, and criteria for inclusion. Furthermore, the separation and compensatory measures based on deficit perspectives blames the individual, creates a structural segregation, and stigmatizes the social status of im/migrant and refugee students as a group.

The implications of this study concern the (re)definition of social integration, recognition of structural obstacles and affordances, and acknowledgement of the relations of power beyond the control of individual. I argue that social integration is not an individual enterprise but a social and collaborative process that needs the affordances of social and structural provisions at the meso-level of organization in schooling and voluntary social arenas. Social integration needs to be redefined as communal and collective processes. This process includes relations of power between individuals, groups and departments that form partnerships for the well-being of im/migrant students, allows for greater mutual reciprocity, and pluralism beyond the confines of neoliberalism’s universal approach to diversity management.
